# Extracting relations from texts using vector language models and a neural network classifier

**DOI:** 10.7717/peerj-cs.1636

**Published:** 2023-10-11

**Authors:** Maksim Shishaev, Vladimir Dikovitsky, Vadim Pimeshkov, Nikita Kuprikov, Mikhail Kuprikov, Viacheslav Shkodyrev

**Affiliations:** 1 Putilov Institute for Informatics and Mathematical Modeling, Kola Science Centre of the Russian Academy of Sciences, Apatity, Russia; 2 Peter the Great St.Petersburg Polytechnic University, Saint Petersburg, Russia; 3 Moscow Aviation Institute (National Research University), Moscow, Russia

**Keywords:** Relation extraction, SKOS, Neural network classifier, Word2Vec, GloVe

## Abstract

The article investigates the possibility of identifying the presence of SKOS (Simple Knowledge Organization System) relations between concepts represented by terms on the base of their vector representation in general natural language models. Several language models of the Word2Vec and GloVe families are considered, on the basis of which an artificial neural network (ANN) classifier of SKOS relations is formed. To train and test the efficiency of the classifier, datasets formed on the basis of the DBPedia and EuroVoc thesauri are used. The experiments performed have shown the high efficiency of the classifier trained using GloVe family models, while training it with use of Word2Vec models looks impossible in the bounds of considered ANN-based classifier architecture. Based on the results, a conclusion is made about the key role of taking into account the global context of the use of terms in the text for the possibility of identifying SKOS relations.

## Introduction

The article explores the possibility of extracting SKOS semantics from general vector models of the language using a classifier based on an artificial neural network (ANN). Automating the extraction of SKOS relations from natural language texts makes it possible to significantly simplify the tasks of constructing applied ontologies, taxonomies, and thesauri, which are widely used in modern intelligent information systems and knowledge-aware applications.

By ‘general’ we mean vector language models built and trained on texts of general vocabulary using “classical” algorithms of distributive analysis. General models during training are not enriched with specific semantics, beyond of distributional one, in contrast to specific models, where word vectors are initially calculated taking into account the known facts of the presence of certain semantic relations between the concepts denoted by words (see, for example, Kim et al., 2016; Naderalvojoud & Sezer, 2020). Despite the fact that specific models demonstrate higher efficiency in various NLP tasks (see, for example, Major, Surkis & Aphinyanaphongs, 2018), the use of general models is still justified, since this does not require time-consuming training of a specific model, including solving the problem of generating high-quality training data of large volumes.

In this article, word embeddings were used as features for training a neural network classifier that allows identifying the presence of ‘broader/narrower’ and ‘related’ SKOS relations between input words. That is, a closed statement of the relations extracting problem is considered, when the type of the discovered relations is known in advance, which allows us to interpret the task of extracting relations as a classification problem. In addition, possible positive effects of expanding the attribute space by components characterizing the context of the joint use of candidate words, as well as components characterizing their morphological (part of speech) and syntactic properties were studied.

The article considered the two most common types of general vector language models: Word2vec (Continuous Skipgram algorithm) (Mikolov et al., 2013) and GloVe (Global Vectors algorithm) (Pennington, Socher & Manning, 2014). In total, eight different English-language models, trained using the indicated algorithms on various general vocabulary data (Wikipedia and Google News data) were experimentally studied. In addition, the results obtained were compared with a classifier trained using an ANN-based contextualized language model with an attention mechanism Bidirectional Encoder Representations from Transformers (BERT) (Devlin et al., 2019). To validate the obtained ANN-classifiers, they were tested on the EuroVoc (Publications Office of the European Union, 2022) and DBPedia (DBpedia, 2022) thesauri. In addition, experiments were carried out on the practical use of the classifier within the “gold standard” for hierarchical relation extraction.

This work continues the research previously described in Shishaev, Dikovitsky & Pimeshkov (2022). The main contributions of our work are as follows:

• Our experiments show that general language models, trained on unlabeled general language texts in the unsupervised learning mode and taking into account the global context of terms can be used to extract relations. The benefit from their use lies in the fact that in this case it is not required to train the specialized language model containing information about the relationships between the terms in a special way, which is a laborious process (requires labeled corpora). At the same time, a simple neural network classifier based on a feed-forward network provides the efficiency of identifying SKOS relations comparable to state-of-the-art results.
• Experimental studies of language models built using various algorithms have been carried out, which have shown that taking into account the global context of terms when building a model is of key importance for the embedding a SKOS-semantics into the model.
• Experiments were carried out with different composition of the feature vector used by the neural network classifier. Experiments have shown that the inclusion of syntactic and semantic features in the vector (in addition to word embeddings) does not give a significant increase in classification accuracy, that is, SKOS-semantics is contained in the language model itself.

## Related Work

To date, a large set of methods aimed at automated extraction of semantic relations from natural language texts has been proposed. In general, existing approaches can be divided into three large groups: (1) rule-based methods; (2) statistical methods; (3) methods based on machine learning. The methods of the first group are based on heuristics that define a certain set of features (usually lexical and/or syntactic) that identify the desired relationship. Usually, this approach is applied to texts of some limited subject area. For example, in Zhang et al. (2010), the authors use syntactic patterns and corresponding rules to extract spatial relations from texts, which are supposed to be further represented as elements of a geographic information system. The aforementioned specialization in a certain narrow subject area determines the main weak point of this approach: poor scalability to texts on other topics.

One example of the implementation of the statistical approach is the subsumption method, which is used to form hierarchical systems of concepts. The method is based on the assumption that “the concept A includes B if the documents in which B occurs are (or almost) a subset of documents in which A occurs” (Sanderson & Croft, 1999):

(1) \begin{eqnarray*}{D}_{KL} \left( A\parallel B \right) -{D}_{KL} \left( B\parallel A \right) \lt T{H}_{N}\end{eqnarray*}

where on the left side of the inequality is a comparison of the mutual conditional probabilities of two terms, and on the right side is a certain threshold of sensitivity or “noise”. Just as in the case of rule-based methods, the statistical approach is based on heuristic assumptions about the statistical properties of data collections that indicate the presence of the desired relationship, which makes it difficult to widely apply them to extract arbitrary relationships between concepts. At the same time, statistical methods impose increased requirements on the amount of available data (observations) in order to obtain statistically significant results. In the context of the task of identifying the presence of SKOS relations between a given pair of concepts considered here, this means that for each pair it is necessary to form and analyze the largest possible corpus of texts that adequately reflect the current ideas about the subject area under consideration and contain lexical representations of the concepts under consideration, which is very labor-intensive task.

Due to significant advances in the field of machine learning, namely, artificial neural networks, methods for extracting knowledge from texts based on ANNs have been most actively developed in recent years. To solve knowledge extraction problems, almost all of the modern machine learning methods is used, the only limitation in this case is the availability of language resources necessary for training models. A review of such methods can be found in many works, for example, in Kumar, (2017), Han et al. (2020), Nasar, Jaffry & Malik, (2021) and Yang et al. (2022). The winning side of machine learning is the versatility of the algorithm used in terms of the type of relations being extracted; the only problem is the formation of a sufficiently large corpus of explicitly or implicitly marked up texts and the choice of a set of features that effectively indicate the presence of the considered relation between concepts. The authors of modern reviews note the use of deep neural networks as the most promising approach to extracting relations based on machine learning. At the same time, significant problems of their application for solving the problem under consideration are also noted: the need for labeled text data corpora, high computational complexity, the impossibility (in the general case) of explaining the principles of decision-making by a trained network and, accordingly, the impossibility of assessing the risk of issuing an incorrect decision by the ANN (Nasar, Jaffry & Malik, 2021). Among other things, it is noted that most methods of extracting relations based on ANN work only with relations presented within the same sentence (Han et al., 2020), that is, they are unable to recognize long-tail relations in the text.

In turn, depending on the nature and way of using a priori information available, various formulations of the problem of extracting relations are possible. The most general form is the task of identifying relations - searching in the text for subsets of pairs of words (lexical constructions in general) linked by similar relations. In this case, the solution is based on clustering a set of samples in some feature space that defines relational similarity (Turney, 2006). Despite the fact that with this approach, clustering does not require preliminary labeling of samples (we are dealing with unsupervised learning), on the next stages, the task of identifying the resulting clusters with one or another subject relation arises—*i.e.,* we have to interpret the obtained relations into the target semantic model, which must be given a priori. In Percha & Altman (2018), for example, the problem of identifying relations between chemicals, genes and phenotypes (how chemicals, genes and phenotypes interact) is solved, and existing manually created databases and thesauri that define subject concepts and relations are used to identify the type of subject relations.

The most modern approaches use the representation of pairs of words as embeddings in a vector space (a classification of different embeddings can be found, for example, here: Jain et al., 2022, the metric of which is interpreted as the semantic proximity of relations between pairs (Turney & Pantel, 2010; Washio & Kato, 2018). The general idea of this approach is to build a specific vector language model in which entities and relationships are all embedded into a common low-dimensional vector space. As a result, for such a language model the equation is fulfilled (≈ - is a sign of approximate equality):

(2) \begin{eqnarray*}{t}_{1}-{t}_{2}\approx {t}_{3}-{t}_{4}\approx r\end{eqnarray*}

where *t*_1_*, t*_2_ and *t*_3_*, t*_4_ –vectors of pairs of terms (concepts) linked with *r* relation.

At the same time, successful examples of building taxonomies by taking into account the mutual context of the use of terms, for example, in the form of a pair–pattern matrix (Snow, Jurafsky & Ng, 2006), allow us to make assumptions about the presence of implicit semantic information in general vector language models, also formed on the basis of the distributive hypothesis.

This assumption is confirmed by the emergence of zero-shot technologies that allow extracting relationships from language models trained on large text corpora using advanced ANN architectures. Such technologies do not require training or fine-tuning of the language model, but they try to extract the relationship between terms directly from the model, assuming the presence of the corresponding prior knowledge in the model. For example, Jain & Espinosa Anke (2022) consider an approach to extracting hypernymy relations from BERT, RoBERTa and GPT2 language models on the base of cloze statements (prompts) and sentence scoring method. A feature of the approach considered in this article is that to extract relations, a specially formulated request to the language model is used, within which two components of the RDF triple are specified—subject and predicate (relation)—and the missing third component (object) is expected in the response.

Our approach also belongs to a similar zero-shot technology, but we use an external neural network classifier to identify the presence of the relationship between terms based on their representation in the language model. In general, zero-shot approaches compare favorably with statistical methods and methods based on specialized language models that embed relationship information in that they do not require the formation and labeling of large training data corpora. On the other hand, compared to query-based approaches, the use of a neural network classifier to identify relationships allows you to fully automate the process of extracting relationships, avoiding manual generation of efficient prompts.

## Materials & Methods

**Figure 1:**
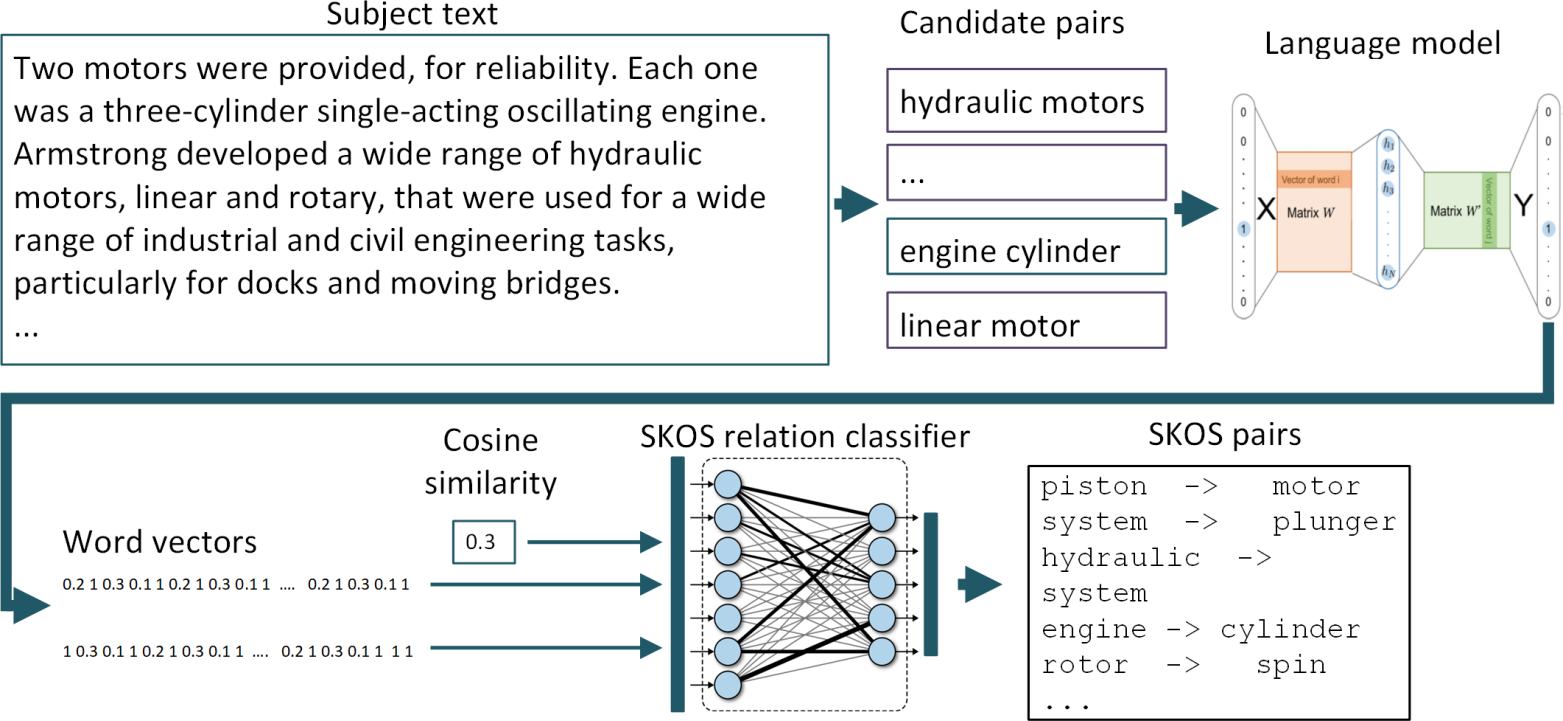
General scheme of extracting SKOS relations using the proposed approach. The figure illustrates the process of extracting the ‘skos:broader’ relation using the example of an encyclopedic article on hydraulic pumps.

The general scheme of the process of extracting SKOS relations from the text for the formation of an applied ontology (taxonomy) using the proposed approach is shown in Fig. 1. At the initial stages, pairs of candidate terms are formed for inclusion in the ontology. Then, using the general language model, vector representations of words are formed, which, along with the calculated cosine distance between them, form a set of features for the classifier. As a classifier, we used a feed-forward ANN trained on labeled data sets. The result of the classifier’s work are pairs of terms, between which the desired SKOS-relation is identified with determined threshold.

Further in this section, the SKOS model, algorithms of the considered language models forming, as well as the method of forming the training dataset, are briefly considered.

### SKOS model

The SKOS model is a widely used framework for building applied ontologies, taxonomies, and thesauri. SKOS has been standardized by the W3C consortium (W3C, 2021) and is widely used in various applications, including engineering. The basic SKOS model includes three types of relationships, defined according ISO 2788 standard (International Organization for Standardization (ISO), 1986) (skos:broader, skos:narrower and skos:related) which makes it an effective means of formally representing taxonomies. The elements of SKOS are classes and their properties, so SKOS defines “a convenient and intuitive map of some subject domain”. In the context of the task of representing taxonomies, the SKOS relation “broader” is of the greatest interest, however, it should be noted that “broader” can denote different paradigmatic relations (“part-whole”, “class-subclass”, “attribute”, *etc*.).

The statement of the presence of a relationship depends on the point of view and the intended scope of the thesaurus. In this regard, in most cases, when identifying the presence of one or another relationship between a pair of concepts, it is impossible to determine the formal function of belonging to a class, the only way to define a class in this case is to explicitly enumerate its instances. Therefore, in our work, we use external reference thesauri (DBPedia and EuroVoc) to train and test the effectiveness of the created classifiers.

The same circumstance determines the main limitation of the proposed approach to extracting SKOS-relations: formally, the accuracy of relation identification will always depend on the point of view of experts. However, the fact that the test of the classifier on an external data source (EuroVoc) that was not used during training showed good results (about 75% accuracy) allows us to speak of some universality of the approach.

Therefore, it cannot be expected that automating the extraction of SKOS relations from the text will make it possible to fully automate the process of constructing a taxonomy, but this will greatly facilitate the solution of the expert’s tasks related to the selection of candidates for inclusion in the taxonomy.

### Word2Vec and GloVe models

As general vector models, in this article we considered models built on various text corpora using the Word2vec and GloVe algorithms. Word2Vec is a family of ANN models using unsupervised learning to create static word embeddings (vector representations). We used language models built using the Skip-gram algorithm: each current word is used as input for a log-linear classifier with a continuous projection layer, and words are predicted at a certain distance before and after the current word. The goal of training a Skip-gram model is to find embeddings that are useful for predicting surrounding words in a sentence or document. More formally, given a sequence of training words *w*_1_*,w*_2_*, …,w*_*T*_ , then the goal of the Skip-gram model is to maximize the mean log probability:

(3) \begin{eqnarray*} \frac{1}{T} \sum _{t=1}^{T}\sum _{-c\leq j\leq c,j\not = 0}\log \nolimits p({w}_{t+j}\mid {w}_{t})\end{eqnarray*}

where *c* is the size of the training context (which may be a function of the central word *w*_*t*_).

Unlike Word2Vec, the GloVe model, in addition to the local context window, uses the global matrix factorization method. This model uses statistical information by learning only on non-zero elements of the word-to-word co-occurrence matrix, rather than on the entire sparse matrix or individual context windows in a large corpus. Thus, language models based on GloVe take into account the broader context of the use of terms. As our further experiments showed, the global context of the term implicitly contains information about the relations of the SKOS-model, which makes it possible to use relatively simple language models to extract them from texts. To train the model, the GloVe algorithm uses a weighted least squares method, which minimizes the difference between the scalar product of two-word vectors and the logarithm of the number of their joint uses:

(4) \begin{eqnarray*}J=\sum _{i,j=1}^{V}f({X}_{ij})({w}_{i}^{T}{\tilde {w}}_{j}+{b}_{i}+{\tilde {b}}_{j}-\log \nolimits {X}_{ij})^{2}\end{eqnarray*}

where *V* is the dictionary size, *w*_*i*_ and *b*_*i*_ are the word vectors and the offset of word *i*, ${\tilde {w}}_{j}$ and *b*_*j*_ are the context word vector and the offset of the corresponding word *j*, *X*_*ij*_ is the number of times word *i* occurs in the context of word *j*, and *f* is a weight function that assigns lower weights to rare and too frequent co-occurrences of words.

### Forming the training data set

**Figure 2:**
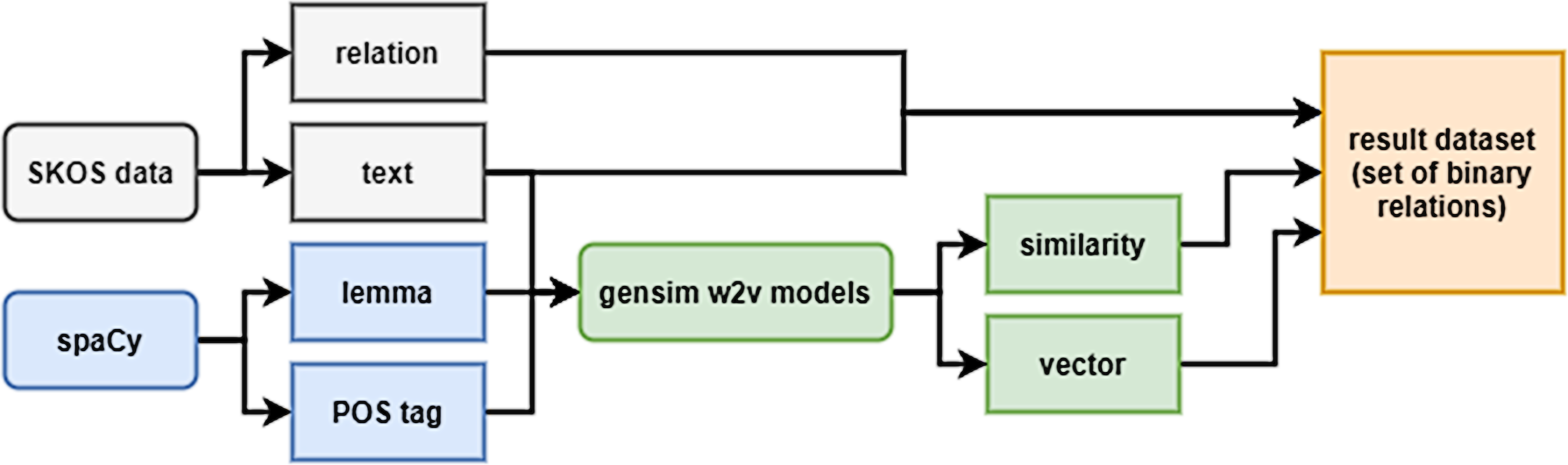
Generalized scheme for creating a dataset.

The training dataset was formed on the base of DBPedia ontology as previously described in Shishaev, Dikovitsky & Pimeshkov (2022). The samples included all triples “concept-relation-concept”, where the concepts are represented by uni- and bigrams (n-grams with *n*  >  2 were not considered, since most of the concepts of the considered ontology are denoted by one- or two-word lexical constructions). For the vector representation of bigrams, averaging was used, as well as the sum of vectors (experiments showed no noticeable difference in performance when using the average or the sum). A generalized dataset formation scheme is shown in Fig. 2.

**Figure 3:**
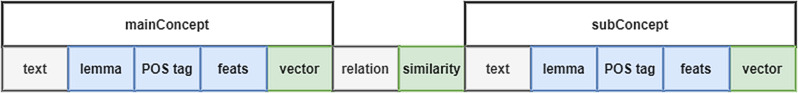
Structure of the sample data.

The extended structure of the sample data is shown in Fig. 3.

 The fields ‘mainConcept’ and ’subConcept’ consists of a list of dictionaries, where each dictionary is responsible for a single word in the lexical representation of the concept (for unigram, this is a list of 1 dictionary). Dictionaries contain the word itself (‘text’ field), its part of speech (‘POS tag’), and their tags (‘feats’) represented by the dictionary. The concepts were tokenized using the spaCy library using the en_core_web_lg (3.0.0) model. Tokens were used to take vectors of individual tokens and then convert them into a concept’s vector.

The ‘relation’ field is a SKOS relation between concepts.

The ‘similarity’ field is a measure of the similarity of vectors, as which a cosine distance or aggregate on its basis was used.

During the experiments, various variants of the training data structure were used. In particular, at the initial stage, to train the basic version of the classifier, only concept vectors, the identifier of the relations between them, and the cosine distance were used. In subsequent experiments, the feature vector was supplemented with morphological and syntactic properties of lexical constructions denoting the concepts under consideration.

To balance the sample, undersampling the majority class was used (equalizing the number of samples of the dominant class with the number of samples of the class of the lowest power). In addition, the set was supplemented with artificially generated samples, including concepts that are not related to the considered SKOS relations (broader and related). An artificial class (‘none’) was formed by randomly combining concepts from the original set (connected by the broader relation) and then checking for non-inclusion in the broader or related class.

## Results

### Basic classifier based on embeddings

In the first series of experiments, the question of the presence of hidden dependencies in the vector language models of the general lexicon was investigated, which makes it possible to identify the presence of broader and related SKOS relations between the corresponding concepts of lexical constructions (uni- and bi-grams) by the values of the vectors of lexical constructions.

The following vector models were considered in the work:

• Two models from the Nordic Language Processing Laboratory’s (NLPL) word embeddings repository - NLPL-5 and NLPL-6, trained on the “English Wikipedia Dump of February 2017” corpus using the Continuous Skipgram algorithm. The dictionary size of the first model is 273,992, the second one is 302,866. The NLPL-5 model, unlike NLPL-6, uses lemmatization of word forms.
• W2V model “GoogleNews-vectors-negative300”. The model is trained on a part of the Google News dataset, contains 300-dimensional vectors for 3 million words and phrases.
• Two models from the Glove project: (1) glove.42B. Common Crawl (42B tokens, 1.9M vocab, uncased, 300d vectors, 1.75 GB); (2) glove.6B. Wikipedia 2014 + Gigaword 5 (6B tokens, 400K vocab, uncased, 300d vectors, 822 MB).

For the listed models, datasets were formed in accordance with the scheme discussed in the previous section. The final sample sizes for each of the considered language models are presented in Table 1.

**Table 1:** Characteristics of training samples.

**#**	**Language model**	**Number of samples for each relationship**	**Total sample size**
1	NLPL-5	20,938	62,814
2	NLPL-6	12,928	38,784
3	GoogleNews-vectors-negative300	17,925	53,775
4	glove.42B. Common Crawl	41,508	124,524
5	glove.6B. Wikipedia 2014 + Gigaword 5	40,690	122,070

On the obtained datasets, various variants of feed-forward ANNs with fully connected layers were trained. Training and testing samples for each dataset were formed on the basis of a 3:1 ratio. Experiments were carried out to train ANNs with different architectures and activation functions. Architecture variations consisted in changing the number of layers from 3 to 6 and using various activation functions - Sigmoid, SoftMax, ReLu. Experiments have shown that variations in the ANN architecture have little effect on the quality of network training, while the key factor in this case is the type of language model used. For this reason, only the most revealing results of experiments are considered below, in which 5-layer ANNs with a funnel 631-500-350-250-150-3 were used, the activation function was ReLU, and training was carried out in 20 epochs.

**Figure 4:**
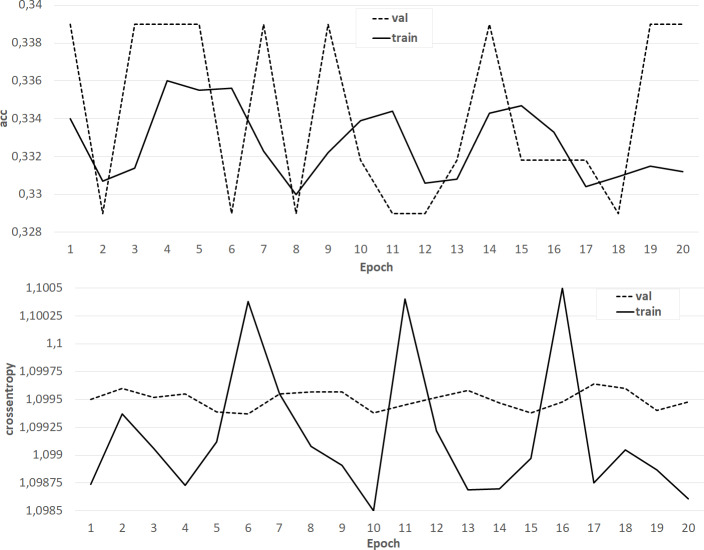
The results of training the classifier on the base of the NLPL-5 model.

**Figure 5:**
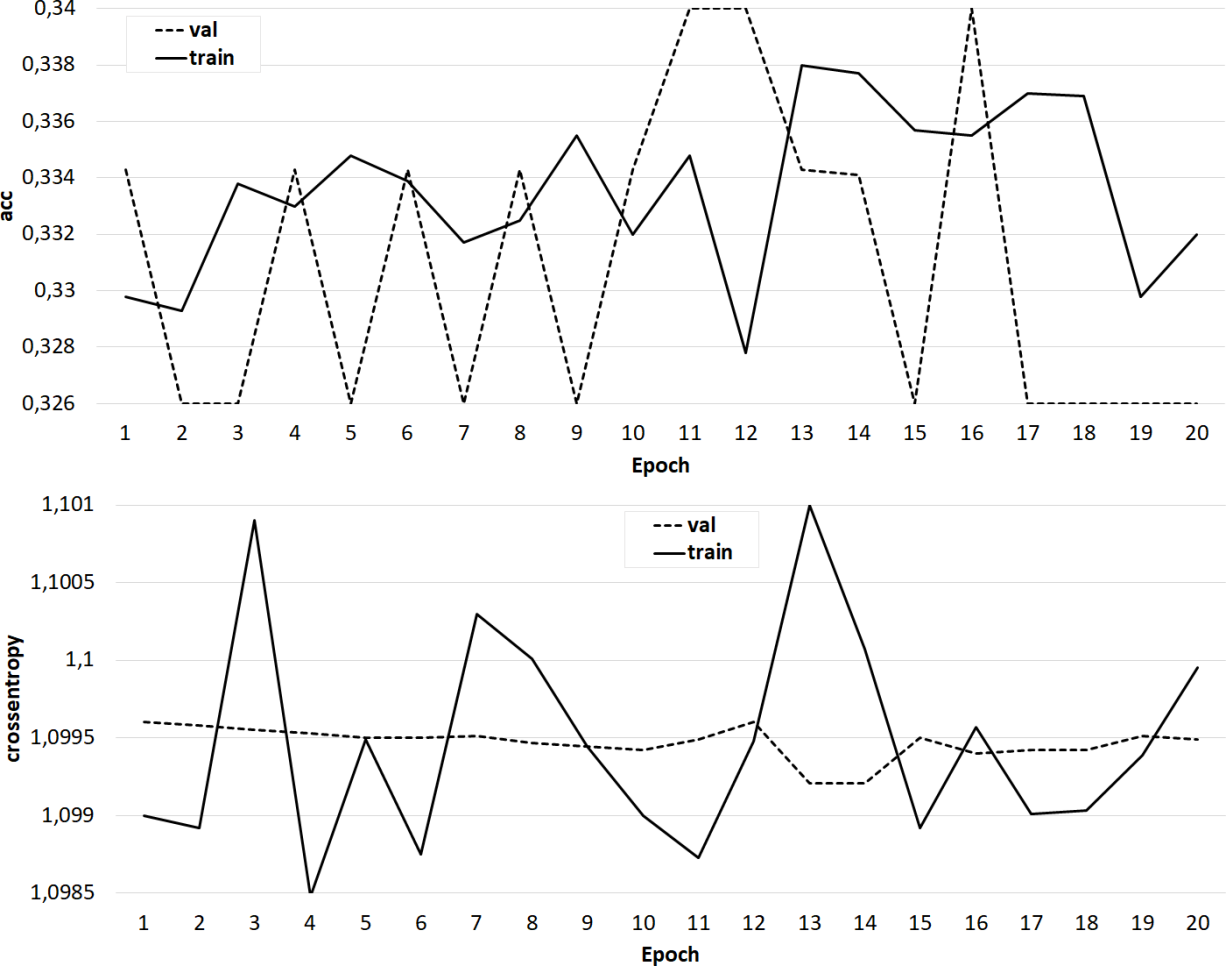
The results of training the classifier on the base of the NLPL-6 model.

**Figure 6:**
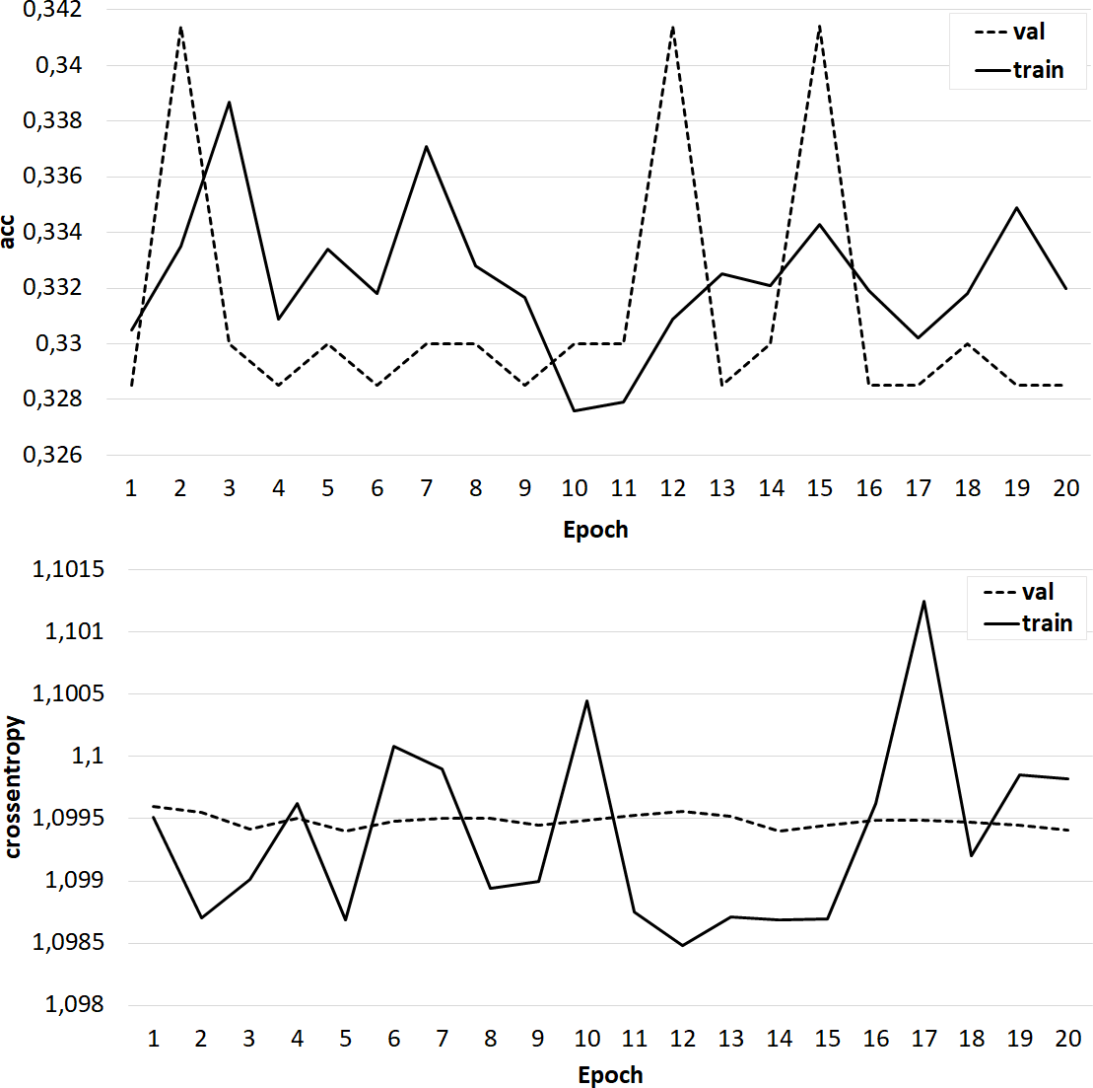
The results of training the classifier on the base of the ‘GoogleNews-vectors-negative300’ model.

**Figure 7:**
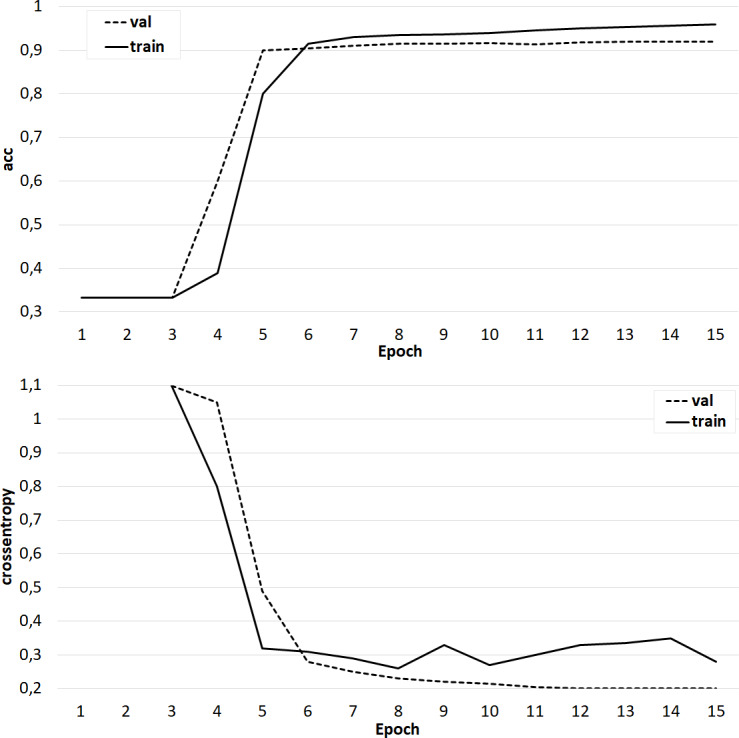
The results of training the classifier on the base of the ‘glove.42B. Common Crawl’ model.

**Figure 8:**
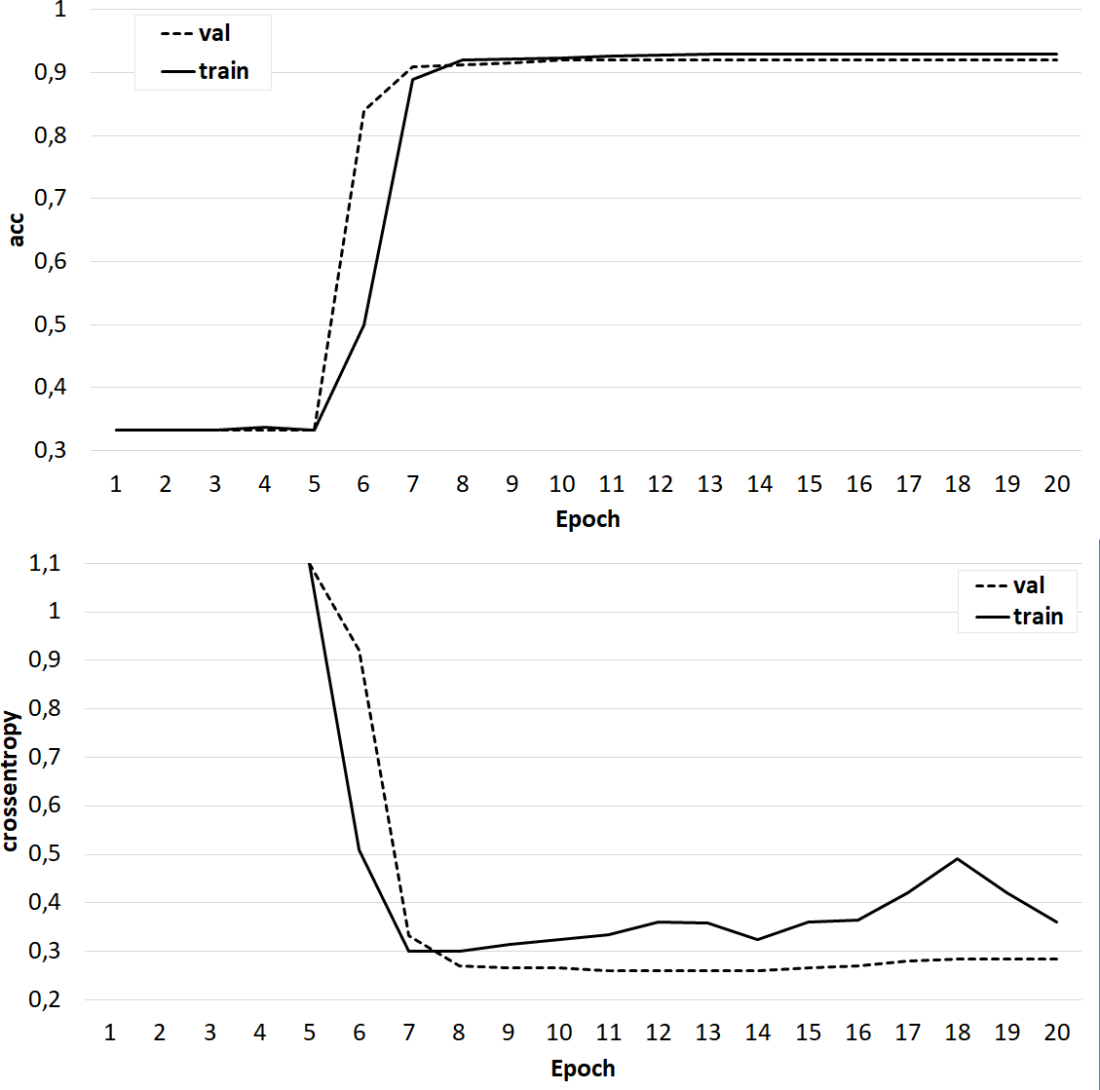
The results of training the classifier on the base of the ‘glove.6B. Wikipedia 2014 + Gigaword 5’ model.

The results of training the classifier in the form of dependences of accuracy and crossentropy on the number of training epochs for each of the five cases are shown in Figs. 4–8. Figures 4–6 present the results of experiments with models built using the Skipgram algorithm (models No. 1–3). During the training process, the accuracy of the neural network classifier does not converge to any stable result, which indicates the probable absence of the desired dependence in the considered language models from the Word2Vec family.

At the same time, classifiers trained on models from the GloVe family showed very good results (Figs. 7–8). In all experiments, a classification accuracy of more than 90% was achieved, while the ANN learning rate did not exceed five epochs. It can also be noted that for a more voluminous language model, the learning rate is higher, which sets one of the promising directions for improving the results. The sharp improvement in the quality of network training observed during the experiments already at the initial stages (within 2 epochs) indicates the presence of a well-identified dependence in the training data.

### Building a classifier based on an extended feature vector

At the next stage of the work, experiments were carried out to assess the influence of syntactic and semantic properties on the accuracy of the classifier. In accordance with the algorithms for constructing the language models considered in the work, the vectors of concepts take into account the context of their joint use only in a positional sense, while the syntactic roles of lexical constructions denoting concepts and the semantics of natural language statements (sentences) are not taken into account, within which concepts are used. The hypothesis in this case was that taking into account the syntactic roles that use a pair of concepts within a single sentence, as well as some common representation of the semantics of such sentences, will improve the accuracy of recognition of SKOS relations. To do this, in subsequent experiments, the feature vector was expanded with semantic images of sentences using a pair of interrelated concepts, as well as their syntactic roles.

The main problem at this stage was the formation of a training data set of sufficient volume. To do this, it was necessary to form a pool of data instances that included a pair of concepts related by the broader or related relationship, as well as examples of sentences containing both concepts at the same time. For the training set of 6,335 unique pairs of concepts connected by SKOS relations used at the first stage, examples of such sentences were found only for 3,340, that is, for about half of the pairs of concepts. Therefore, within the framework of this series of experiments, the transfer learning technique was also used to form a larger training set. As a source of training data, we used the Leipzig corpus (Goldhahn, Eckart & Quasthoff, 2012), which contains a set of the most frequent pairs of commonly used terms with examples of English sentences that include them. Pairs of concepts were selected from the Leipzig corpus, identified by a previously trained classifier as being in one or another SKOS-relationship with a threshold of 0.95. As a result, an expanded training data set was obtained, including 63,349 data instances (16,725 related, 6,523 broader, 40,101 empty).

**Figure 9:**
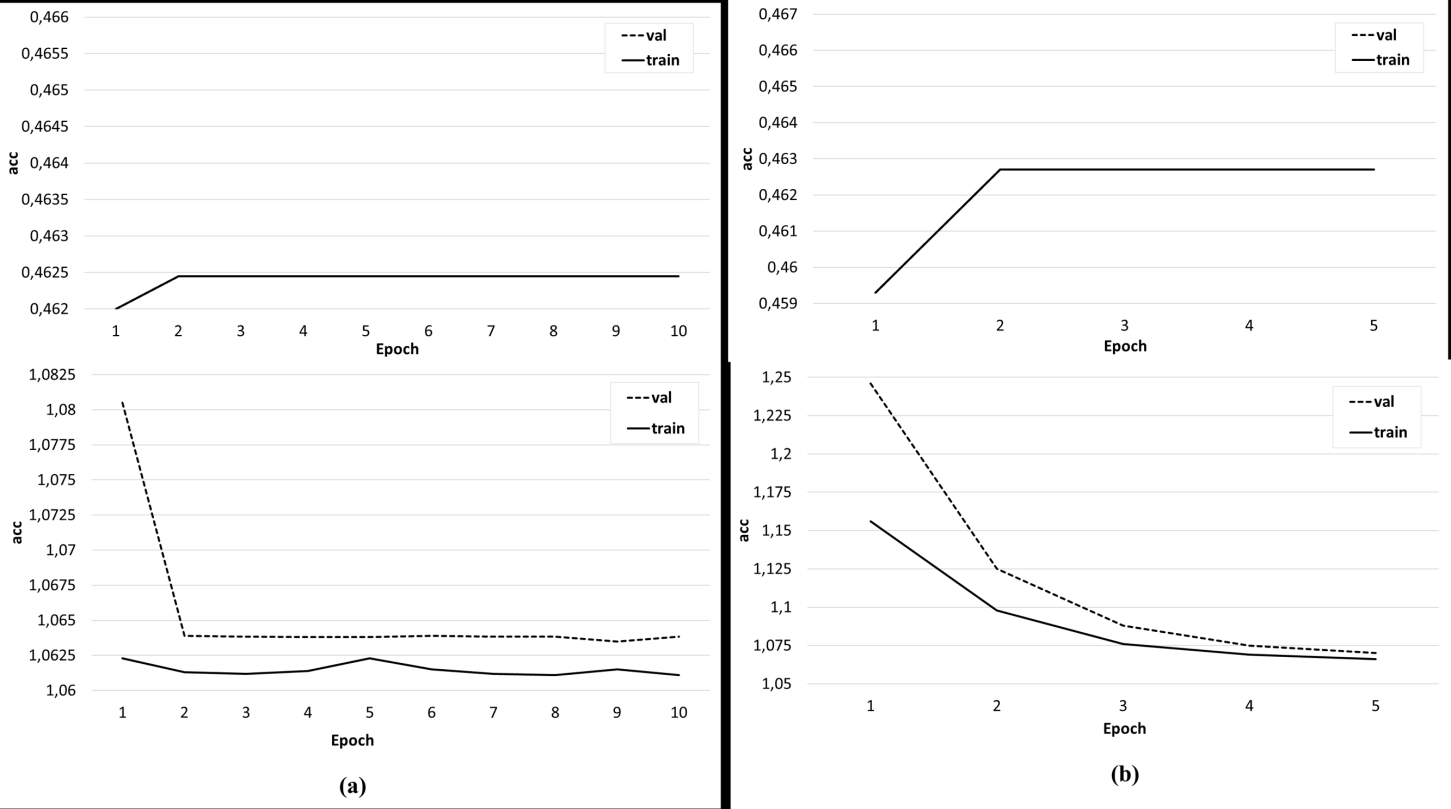
The result of training the classifier (A) on the original data set and (B) with an extended feature vector.

**Figure 10:**
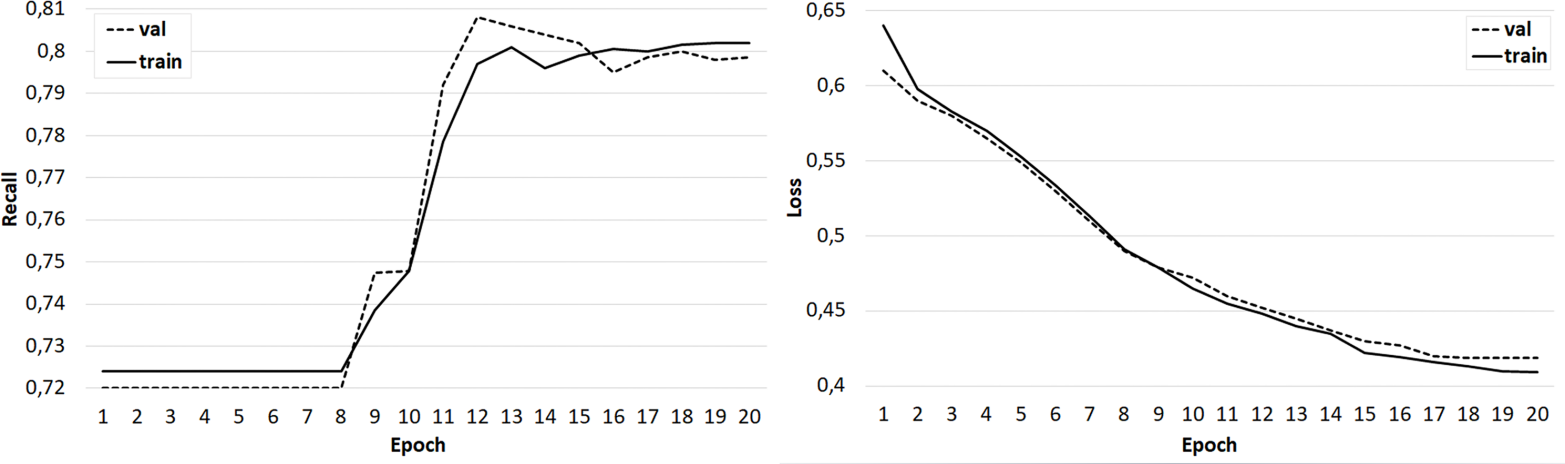
The result of training the classifier with a feature vector extended by the syntactic and morphological features of candidate words.

Several sets of experiments were carried out using different variations of the training sets. In the first series of experiments, the effectiveness of the three-class classification (broader/related/none) was considered. The classifier was trained on the original training set, where the feature vector included only candidate word vectors, as well as an identical set, but with a feature vector extended by the semantic image of the sentence, including the concepts under consideration. The Doc2Vec algorithm (Le & Mikolov, 2014) was used to obtain the semantic image of the sentence. Experiments have shown that the addition of a feature vector with a semantic image of a sentence does not improve the accuracy of classification, but positively affects the learning rate of the classifier (Fig. 9). In the next series of experiments, a two-class classification was considered. In the third series of experiments, using the original data set, the feature vector was expanded with the syntactic roles of the dependent and main words, as well as their part-of-speech features. In this case, the accuracy of the classifier is significantly higher (about 80%) and is comparable to the accuracy of the original classifier (Fig. 10).

### Evaluation of effectiveness of the classifier

When testing the quality of the classifier, it is important that the test data be taken from practice, since this to some extent solves the problem of the lack of a general understanding of semantics. The use of actually used data (external thesauri) to check the model provides an opportunity to assess the quality of the result, at least within the framework of the tasks that these thesauri are oriented to. At the same time, we understand that a universal model that reflects all possible interpretations of semantics cannot be created (Bakarov, 2018). To test the original classifier trained on DBpedia data using a simplified feature vector, a training set was generated containing the SKOS concepts of the EuroVoc thesaurus. Terms were vectorized using the glove.42B.300dB model. Results of evaluation:

• a total of 21,221 out of 21,453 were identified (98.92%);
• ‘broader’ matches: 4,932 out of 5,840 (84.45%);
• matches ‘related’: 236 out of 9,942 (2.37%);
• ‘related’ is defined as ‘narrower’: 205 out of 5,671 (3.61%).

The cardinal difference between the results when using the Word2Vec and Glove models for training the classifier suggests that the accounting in the corresponding embedding the global context of use of the concept has a decisive influence on the quality of the classification. At the same time, modern research shows that context is best taken into account in ANN architectures that use the attention mechanism (Bahdanau, Cho & Bengio, 2016). In turn, the language model that implements this mechanism and demonstrates outstanding results in a wide range of NLP tasks is BERT. In this regard, in order to test this assumption and compare the results with the case of using the contextualized language model for training the classifier, a BERT classifier was trained on the best dataset. The initial BERT model was used—104 languages, 12-layer, 768-hidden, 12-heads, 110M parameters—which was fine-tuned on a labeled sample of 7,000 instances: 3,500 pairs of words associated with SKOS, and 3,500 random steam. The resulting BERT classifier provided an accuracy of 93% on the test sample, which corresponds to the accuracy of the original classifier based on the feedforward network.

### Comparison with state-of-the-art results

In open sources, there are no recent works devoted to the extraction of SKOS relations from texts. One of the works where this task is considered is dated 2011 (Wang, Barnaghi & Bargiela, 2011), it proposes a technology for extracting SKOS relations from unstructured text corpus based on probabilistic topic models. The best precision of relationship extraction obtained by the authors was 86.6%. However, the assessment of the correctness of the revealed relationships in this work was carried out manually, with the involvement of experts, which excludes the possibility of a quantitative comparison of these results with alternative approaches.

In this regard, to compare our results with analogues, we used the “gold standard” test of the Taxonomy Extraction Evaluation (TExEval) set (Arabic Language Technologies (ALT), 2023) contains hyponymic-heteronymic asymmetric relations, which are a subspecies of a hierarchical relationship indicating subordination between two terms. The gold standard relations were collected from WordNet and other well known, openly available manually constructed taxonomies, classification schemes and/or ontologies. The package contains a list of English terms from a manual gold-standard taxonomy rooted on “food” and a list of terms rooted on “science”.

For evaluation, balanced test datasets were formed by adding deliberately false pairs. To do this, the first word in all pairs was first replaced by “food”, then by “science”. A previously trained three-class (broader/related/none) classifier was used to identify the relationship of hypernymy within the test. The classifier output values were normalized, after which the presence of one of the three relations was identified by the maximum value of the corresponding output. Relations between terms obtained using the proposed classifier have been evaluated against collected gold standards using standard precision, recall and F1 measures. In our case the listed measures reach 52.8, 32.7 and 40.4%, respectively, for the food-rooted dataset and 54.8, 29.7 and 38.5% for the science-rooted dataset.

Table 2 compares the effectiveness of the approach considered in this article (FF-classifier) with other models tested on science-rooted and food-rooted datasets from the TExEval corpus:

**Table 2:** Model efficiency comparison.

**Model**	**Food-rooted**			**Science-rooted**		
	P	R	F	P	R	F
TAXI	13.2	25.1	17.3	35.2	35.3	35.2
Graph2Taxo	n/a	n/a	n/a	84.0	30.0	44.0
MLM/LMS	25.2	24.6	24.9	39.3	36.7	37.9
FF-classifier	52.8	32.7	40.4	54.8	29.7	38.5

**Notes.**

Abbreviations P precision R recall F F-score

•a taxonomy induction method “TAXI” based on lexico-syntactic patterns (Panchenko et al., 2016);

•Graph2Taxo model based on graph neural networks (Shang et al., 2020);

•MLM/LMS model proposed in Jain & Espinosa Anke (2022), which implements a zero-shot approach using pretrained language models (BERT, RoBERTa and GPT2) and the prompting and sentence scoring technique.

We can see that the proposed approach outperforms in terms of precision (P), recall (R) and F-score (F) some of the existing approaches and is comparable in efficiency with state-of-the-art results.

## Conclusions

The conducted studies and experiments in general allow us to give a positive answer to the question of the possibility of extracting SKOS relations from natural language texts using a neural network classifier trained on general vector language models. In particular, the following conclusions can be drawn from the results of the work:

• The fundamental difference in the quality of the work of the neural network classifier, depending on the vector language models used—Word2Vec or GloVe—suggests that the determining factor is the consideration of the global context of the use of the term when forming the language model. Accounting for the global context is typical for the GloVe model learning algorithm, while in the Word2Vec model generation algorithms only the local context is taken into account—the average window size when training the model does not exceed 5–7 neighboring words. This assumption is also confirmed by the results of experiments on classifier training using language models built using BERT technology.
• Equally good results for the GloVe 6B and 42B models, which differ in vocabulary size by a factor of seven, also leads to the conclusion that the size of the model in this case is not critical.
• The good result of identifying SKOS relationships on data different from those used in training the original classifier (the EuroVoc thesaurus) suggests that the classifier can be used on arbitrary English texts (not only on the subset of the language presented in DBpedia).

As noted earlier, the skos:broader relationship is too generic to be used directly in taxonomies. Therefore, the actual direction of further work may be to study the possibility of extracting more semantically loaded relations from the general vector models of the language, such as “part-whole” or “class-subclass”.

## Additional Information and Declarations

### Data Availability

The following information was supplied regarding data availability: The data and code are available at Zenodo: Vladimir Dikovitsky. (2023). Dikovitsky-Vladimir/SKOS: Added SKOS classifier with sentences (Version v2). Zenodo. https://doi.org/10.5281/zenodo.8220330. The description of the content is available in the README file at GitHub: https://github.com/Dikovitsky-Vladimir/SKOS/blob/main/README.md.
